# The extent of child sexual abuse in Botswana: hidden in plain sight

**DOI:** 10.1016/j.heliyon.2020.e03815

**Published:** 2020-04-26

**Authors:** Nankie M. Ramabu

**Affiliations:** Leeds Beckett University, City Campus Leeds, LS1 3HE United Kingdom

**Keywords:** Psychology, Child sexual abuse, Botswana, Teenage pregnancy, Extent of CSA, Secondary data, Child protection

## Abstract

**Introduction:**

Child Sexual Abuse (CSA) is a worldwide persisting public health problem which has generated interesting discussions within child protection scholarship. Globally as well as in Botswana, CSA estimates prove challenging to establish. This study sought to establish the extent of CSA in Botswana by use of existing data as well as narratives from key informants.

**Methods:**

CSA existing data was extracted from the Botswana police services records and Botswana statistics for the year 2013, 2014, 2015 & 2016. In-depth interviews, semi-structured interviews, were used to collect data from policymakers, child protection practitioners, and caregivers respectively. The study sites were Gaborone city and Letlhakeng village. Qualitative data were analysed using NVivo qualitative data analysis computer software. Whereas the quantitative data I analysed using the excel Microsoft office 365.

**Findings:**

According to CSA existing data, in 2013 defilement among children was 97 (0.2%). Whereas 901 children were reported pregnant in 2013, almost ten times more than defilement cases. In the same year, there was a high number (1058) of children who stayed away from school. Participants' narratives reported CSA to be an escalating problem in Botswana.

**Conclusion:**

The finding that teenage pregnancy statistics are higher than defilement statistics needs further research to categorise and inform child sexual abuse programming in Botswana.

## 1 Introduction

### 1.1 Overview

Child sexual abuse is a worldwide persisting social and health problem which has generated interesting discussions among child protection scholarship. In Sub Saharan Africa, CSA became a topic of discussion in the 1980s and 1990s, with South Africa more advanced than its neighbouring countries (Bowman and Brundige, 2014). A reported incest case documented in Schapera (1941) is so far the earliest CSA case in Botswana. Therefore, in the absence of a clear historical account of CSA in Botswana that could have been crucial in establishing CSA as a social problem in Botswana, one could say that international legislations such as United Nations Convention on the Rights of the Child (UNCRC, 1999), Human Rights, Additional Protocol on the Sale and Procurement of Children brought CSA to public attention as a social problem needing intervention. Additionally, behavioural studies in the context of HIV/AIDS have brought gender-based violence to public attention, with children found to be susceptible to HIV infection (Underwood et al., 2011; Phaladze and Tlou, 2006; Phorano et al., 2005; Decker et al., 2015). Further, the Botswana media also plays a key role in bringing CSA to public attention.

In 2018, UNICEF Botswana together with Botswana government launched a campaign called Eseng Mo Ngwaneng loosely translated to imply touch not the child. This campaign led by the First Lady Neo Masisi, intended to sensitise the Botswana society to collectively take responsibility in the safeguarding of CSA. This Eseng Mo Ngwaneng social mobilization emanates from Botswana Violence Against Children Study (VACS) which showed that children in Botswana experience sexual violation of some sort.

### 1.2 Suppression of CSA

It is important to acknowledge that there could be reasons why CSA has not yet received much attention in Botswana. This could be due to other competing public health priorities affecting the country such as HIV/AIDS (UNICEF, 2011), poverty alleviation strategies (Botswana Ministry of Local Government Report, 2008) and addressing basic needs of Orphans and Vulnerable Children. Another factor that could serve to conceal CSA which has a cultural dimension could be, socialisation of children to be submissive and keep silent about issues even those that could negatively affect them (Lalor, 2004). Additionally, little attention has been paid to sexual abuse perpetrated by other children, and commercial sex exploitation of children (Policy Maker, Social Services). In fact Botswana has been reported to be a source and destination for trafficked women and children, some of which have been trafficked for sexual exploitation (http://www.refworld.org). Other child sexual abuse acts which are of interest in this paper are teenage pregnancy and child marriage. Botswana like other developing countries is plagued by children dropping out of school due to pregnancy (WHO, 2014; UNICEF, 2016). However, these acts are usually not considered alongside the CSA statistics, something that this paper seeks to explore.

### 1.3 Definition of CSA

Though there is yet to be a universal definition of CSA, this paper adopts World Health Organization definition (WHO, 2017: vii) which defines CSA as “*The involvement of a child or an adolescent in sexual activity that he or she does not fully comprehend and is unable to give informed consent to, or for which the child or adolescent is not developmentally prepared and cannot give consent, or that violates the laws or social taboos of society”*. The WHO emphasises that children also perpetuate sexual abuse against other children and should be addressed. Botswana Children's Act 2009 made a provision for the protection of children against sexual abuse as it purports that *“every child has a right to be protected from sexual abuse and exploitation, including prostitution and pornography (Botswana Children's Act, 2009, A.57, section 25(1)).* The Act further mentions sexual abuse under harm as an act inflicted deliberately on a child and assaulting a child; sexually abusing a child or allowing a child to be sexually abused (part 1c). The following offences in section 57 of the Children's Act 2009 are defined as corruption of children and elaborated as; (1) a parent, other relative or other person having custody of a child who-

(a) Induces, coerces or encourages any child to seduce any person or to engage in prostitution;
(b) Induces, coerces or encourage any person to seduce, prostitute or cause the seduction, or prostitution of any child;
(c) Induces, coerces or encourages a child to commit any other sexually immoral act;
(d) Abducts a child or induces, coerces or encourages any person to abduct a child for the purpose of corrupting the child in any manner referred to in this sub-section;
(e) Induces, coerces or encourages any person to cause a child or any other person to do any act referred to in this subsection shall be guilty of an offence and shall be sentenced to a fine of not less than P20000 ($2000) but not more than P50 000 ($5000), or to imprisonment for a term of not less than two years but not more than five years, or both.

Additionally, section 58 of children's act discourages anyone to cause children to watch or involve in the making of pornographic materials. Section 59 indicates that *"no person shall encourage, force or allow a child to cohabit with the person in a relationship of a sexual nature*."

### 1.4 The extent of CSA

The extent of CSA is still challenging to determine. Pereda et al. (2009) compared Finkelhor (1994) prevalence of CSA study with thirty-eight prevalence studies from 21 countries. In Pereda et al. (2009) meta-analysis, it showed that CSA is still a widespread problem in the society ranging from 0-53% for men and 0–63% for women. Pinheiro (2006) in the violence against children study carried out in over sixty countries show that over 220 million children are sexually abused-150 a million girls and around 73 million boys. Stoltenborgh et al. (2011) meta-analysis of CSA prevalence places global CSA prevalence at 18% for girls and 7.6% for boys. The WHO (2017) global estimates based on Stoltenborgh et al. (2011) show around 18% of girls and around 8% of boys reported they suffered contact sexual abuse. Whereas, Barth et al. (2013) meta-analysis of CSA prevalence estimates 8–31% of girls and 3–17% of boys globally, experience some form of contact sexual abuse. In Botswana, the 2016 national VACS study shows that 9.3% of females and 5.5% among the 18–24 year olds in Botswana experienced sexual violence.

### 1.5 Causes and consequences of CSA

There is an attempt by scholars in CSA scholarship to develop a comprehensive framework to determine causes of CSA. Mustaine et al. (2014) in their study on community characteristics and child sexual assault, identified that economic hardship, housing density and presence of a registered sex offender were positively associated with child sexual assault. These findings by Mustaine et al. (2014) resonate with some findings from UNICEF, Save the child & World Vision (2012) that suggests main causes of CSA in Botswana include rapid social change, poverty, HIV/AIDS, male-dominated social structures, cultural practices, transactional sex and weakening family structures. These factors show that numerous child protection challenges remain amidst Botswana undergoing rapid economic growth and enjoying upper middle-income status.

With regards to CSA consequences, this paper chooses not to dwell on the consequences. This is with the view that CSA is wrong with or without consequences. However, one could allude to that, popular discourse link CSA to mental health (Hanisch and Moulding, 2011). The link between Child Sexual Abuse and mental health has led to therapeutic enterprise developed to address CSA. Though some CSA victims might not have psychological effects, they could be forced to define themselves with the dominant discourse of treatment interventions (Woodiwiss, 2014) and therefore, could fail to contextualize individual experiences.

### 1.6 Rationale

There is a paucity of literature on Child Sexual Abuse in Botswana. Health programmes in Botswana have largely avoided the needs of children who have been sexually abused, as such programmes seek a wider focus on reproductive health and gender-based violence in adults. Most of the studies on children and sexuality are in the context of HIV/AIDS and gender-based violence (Shannon et al., 2012; Seloilwe and Thupayagae-Tshweneagae, 2015; Alao, 2006; Rakgoasi and Odimegwu, 2013) and focusing primarily on adolescent girls. Therefore, there is an urgent need to explore CSA in a country (Botswana) with a high prevalence of Human Immunodeficiency Virus (HIV) (BAIS III, 2009; BAIS IV, 2013). Conceived at the backdrop of this as well as building on recent developments in child protection (United Pinheiro, 2006; Botswana Children's Act, 2009; Sloth-Nielsen, 2012; WHO, 2014; UNICEF, 2013; Union, 2016), this study determines the extent of CSA in Botswana. This paper is based on my doctoral thesis, which sought to determine how Botswana's Child Protection System safeguards children from sexual abuse. Using the existing data from Botswana national records, the paper seeks to answer the question; what is the extent of CSA?

## 2 Methodology

Despite the ambiguity of Mixed Method Research (MMR) expressed by some scholars (Zachariadis et al., 2013; Creswell, 2009; Östlund et al., 2011; Teddlie and Tashakkori, 2006), this study employed MMR that is defined by Creswell (2013: 244) as "*an approach to inquiry that combines both quantitative and qualitative forms of research. It involves philosophical assumptions, the use of qualitative and quantitative approaches, and the mixing or integrating of both approaches in a study”.* The study was predominantly qualitative with the qualitative playing an auxiliary role. Triangulating methods was that, qualitative data could contextualize the quantitative data by enriching understanding and allowing for new or deeper dimensions to emerge (Jick, 1979); answer different research questions; explore the phenomenon under study in depth and from different perspectives. The qualitative strand of MMR served to increase cultural sensitivity since it captured the participants' lived experiences.

Qualitative data from primary data sources which included (1) in-depth interviews with program implementers at national-, district-, and service delivery-levels; 2) semi-structured interviews with caregivers. A total of 26 key informants, 3 semi-structured interviews were conducted. Whereas the quantitative strand, subjected child sexual abuse and teenage pregnancy existing data to analysis. The data for 2013, 2014 and 2015 were obtained from Botswana Police services records and Botswana statistics office. All these data were national-level data, aggregated in printed reports. This limited the extent to which the data could be subjected to statistical analysis.

The in-depth interviews were purposively sampled to focus on participants that were knowledgeable about the child protection system. Nine interviews (in-depth and semi-structured) were conducted in Letlhakeng village, and fourteen interviews (in-depth and semi-structured) were conducted in Gaborone city. Deductive approach was utilised to analyse qualitative data using the NVivo data analysis computer software (Bazeley & Jackson, 2013). NVivo has been shown to have inbuilt reliability procedure (Bergin, 2011). The quantitative data were analyzed using excel to produce graphs for easy presentation.

## 3 Ethical considerations

Since this was a Doctor of Philosophy research study, ethical approval was obtained from Leeds Beckett University Institutional Review Board, research ethics application project no 304. The research was conducted in Botswana and therefore, obtained ethical approval from the Human Research Documentation Committee (HRDC), the ethical clearance reference number PPME-13/18/1 vol VIII (470). For the data to be collected among village research participants, assent was sought from the Kgosi before recruiting community members to participate in the study (Ramabu, 2019). All participants agreed to participate in the study and therefore, signed a written consent to participate. Due to the sensitivity of the subject under study as well as the discomfort of the study participants to be audio-taped, only 3 in-depths interviewees consented to be audio-taped. The audio-taped data were then transcribed. As part of adhering to Botswana ethical consideration, the research findings were disseminated to Botswana community and policy makers. To disseminate to the policy makers, a one day dissemination workshop was conducted with policy makers. Dissemination to the community occurred through Botswana Radio Station (RB1) in Setswana language. This radio station was targeted because it reaches all parts of Botswana.

## 4 Reflexive research

My perspective on this study was informed by my position as a black native from Botswana with South African parents (South African language and Botswana culture), having worked as a Child Nutrition Practitioner, a Nutrition Policy Maker, an Intercultural Researcher, a victim of domestic violence as well as being a survivor of CSA. Additionally, I have studied and lived part of my adult life in England. That is, I carried out this research and interpreted findings through the lens of these multiple identities. It is against the backdrop of my historical, political and economic experiences that the study findings could be understood. Berger (2015) argues that reflexivity is affected when a researcher has an insider status and share participants' experiences. According to Berger (2015), this insider status could serve to overlook and normalize social ills. On these bases, I acknowledge that my multiple identities could mediate this study. However, an insider position is seen as an advantage in giving me access to information that might not be well understood from an outsider's perspective.

## 5 Findings

### 5.1 Defilement

**Figure 1:**
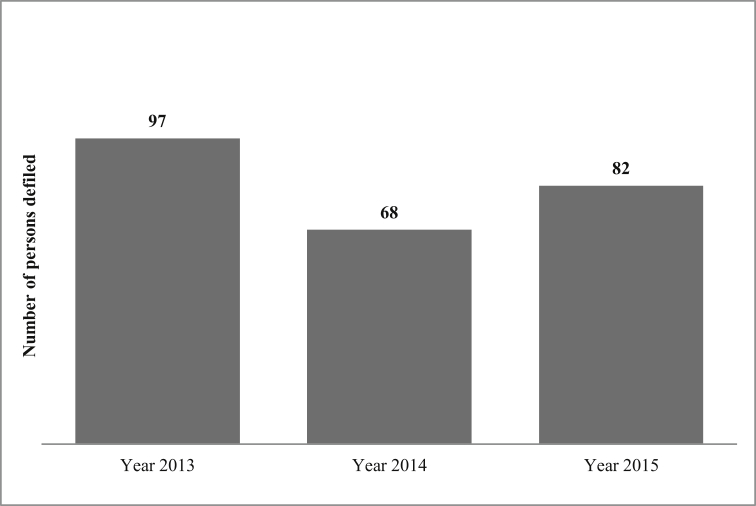
National level defilement of persons under 16 years.

Based on the secondary data, all defilement cases were for persons under the age of 16 years. In these defilement cases, there were no boy victims of defilement. There were 4 incest cases reported in all the three years. However, incest cases were not included in this analysis because the age category of subjects was unclear. According to Figure 1, defilement cases slightly decreased between 2013 and 2014; while slightly increasing between the years 2014 and 2015. The extent of CSA in 2015 is estimated as the number of defilement cases/the population of children under 16 years∗100%=(82/500 000)∗100% = 0.02%.

### 5.2 Rape

**Figure 2:**
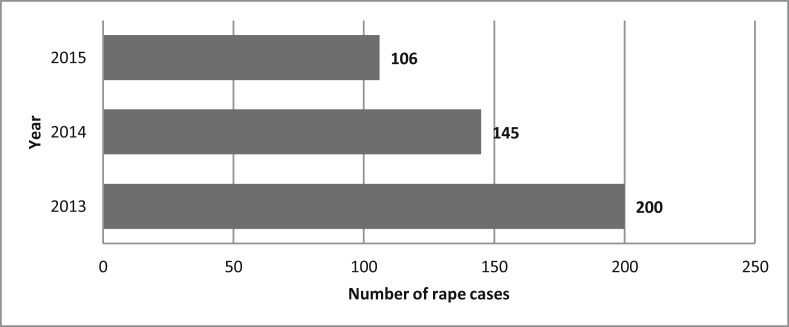
National level rape cases for persons over 16 years.

With regards to rape, data extracted were for persons over 16 years. Almost all rape victims were female, with only one rape victim in the three years being male. These data indicate that reported rape cases declined over the three years. The highest decline observed was attained between the year 2014 and 2015. Due to the absence of other variables such as perpetrator and victim profiles, data were not further analyzed as intended. Figure 2 presents the number of rape cases nationwide in the 3-year period.

The 2013–2015 reported defilement and rape trends were compared to the empirical data from ten years ago which were also derived from Botswana Police Services (Smith, 2008). Table 1 illustrates defilement, rape, and incest of children and adults in Botswana in 2003, 2004 and 2005.

**Table 1:** 2003–2005 Reports of incidents of CSA and rape from Botswana Police Services.

Year	2003	2004	2005
Rape			
Under 10 years	49	63	61
11–17 years	316	418	294
18 years and upwards **(Total)**	923 **(1288)**	911 **(1392)**	987 **(1342)**

Defilement			
Under 10 years	33	14	23
11–16 years **(Total)**	230 **(263)**	100 **(114)**	266 **(284)**

Incest			
Under 10 years	1	0	0
11–17 years	1	0	1
18 years and upwards **(Total)**	1 **(3)**	0 **(0)**	2 **(3)**

Source (Smith, 2008).

The table illustrates that reported rape of children under 10 years, slightly increased over the three-year period. Whereas reported rape of children 11–17 years, increased from 2003-2004 and then decreased between the years 2004 and 2005. At the time of this data, Botswana general population was estimated to be around 1.5 million (Botswana Population Census, 2011). The child population in 2001 was estimated at 40% of the general population, which was around 600 000 children. From this, the data show that in 2005, the proportion of children 11–17 years (21.91%) who were raped were more than that of the proportion of children under 10 years (4.55%) who were raped; second: The proportion of women 18 years and above who were raped (73.54%) was a little over threefold that of children 11–17 years (21.91%) who were raped. The proportion was calculated by dividing the number of persons within an age group by the total number of reported cases of rape and multiply that answer by 100.

**Figure 3:**
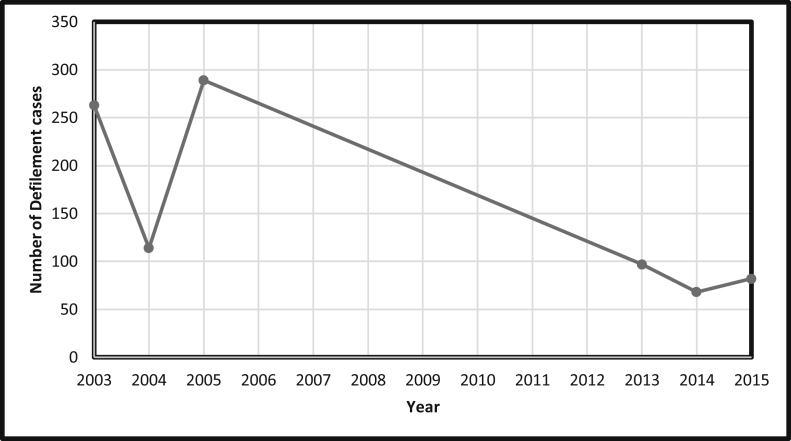
Nationwide number of defilement of children under 16 years.

Lastly, incest cases were rare with 6 cases reported in the period of 2003–2005. When 2003–2005 and 2013–2015 data were compared, the trend shows that the defilement cases decreased over the years. This decline in defilement cases over the years should be considered alongside the teenage pregnancy findings presented later in this section (please Figure 3 below).

Though the extent of CSA could not be well established through the existing data, Participants' narratives reflect CSA to be a problem. As some participants indicated below;

*It is a problem. Children as young as 3–5 years are being assaulted, mainly girls and male perpetrators. The assault on boys is a new phenomenon and in the early stages of discovery*– Prosecutor, Gaborone City.

*The abuse of boys is also the new thing coming in. It is on the rise. The children when they explain to parents, you know parents do not understand same sex, which is men having sex with other men. You know straight away, they will cut it off and dismiss this child*- Gender Officer, Civil Society

How children dress is reported to be perpetuating abuse as one participant indicated *"there was a case where a child was abused, and the authorities said; what was she doing not dressed properly? It just became a joke. These are some of the issues that are a serious concern. Like we are saying Police Officers are not child-friendly".* – UNICEF, Botswana.

In addition to child sex offenses perpetrated by adults, child to child sexual abuse is reported to be a growing problem. As one participant indicated;

*It is a problem. Last year we had about 6 cases which involved girl children and 1 case which involved a boy. Amongst the cases; 2 cases involved children above 12; 4 cases involved children who were below the age of 12 [7, 8 and 9 years]. Most of the cases were perpetrated by children-* Police Services, Gaborone City.

Whereas Participants' narratives reflect children left to self-care, perpetuating risky behaviors that could lead to children engaging in sexual activities. As one participant narrated that; *"Children nowadays do unbelievable things, and I was so shocked. Our children nowadays seriously like sex. I don't know if it is the food they eat or whatever gives them libido. These children are aged from eleven years. They were telling us that when they say sleep over, you will find out that the parents are not there. They will close everything and stay in the house and inhale marijuana and have sex. So these kids are complicated too. It is no longer adults, even among themselves. Sexual abuse among children is not something that gets handled by the law"*- OVC Technical Officer, Civil Society.

Further, Participants' narratives from Letlhakeng village reflect alcohol, poverty, and failure to prosecute perpetrator to be perpetuating Child Sexual Abuse.

*Child Sexual Abuse is a problem because most of the villages are settlements and the parents drink [alcohol] and therefore remove children from school* – Police Services, Letlhakeng Village.

*It [Child Sexual Abuse] is a problem. Parents in Letlhakeng are not taking care of children primarily due to alcohol. When the child is found to have been sexually abused, the parents don't come to support the child. Alcohol is the main thing perpetuating the problem with parents not taking care of children. Other issues include poverty and the challenge of perpetrators not penalized for abusing children* – Primary School Guidance and Counselling Teacher, Letlhakeng Village.

*Some community members come to report; some do not come forth to report cases. I worked a long time in Phuduhudu (a settlement in Letlhakeng village) from 2002 to 2011. Some parents in that settlement used their children as a source of income. I used to address kgotla meetings to sensitize the community* – Police Services, Letlhakeng Village.

Whereas in Gaborone city, pedophilic behavior, under reporting among others were reported as perpetuating Child Sexual Abuse. As reported by some participants below;

*I am currently having three cases, one prominent and of a pedophile who opened a tutoring school for children and known to be sexually abusing the children. He is of French origin and has been here for a while*- Gender-Based Violence Activist, Gaborone City.

*It is a big problem. The challenge is that parents don't report* – Caregiver, Gaborone City.

*CSA is a problem. There is an issue of under-reporting due to parents refusing to take cases up. So far the suspected cases are not many, but that doesn't mean that they [children] are not getting sexually abused. It is the nature of the community that we don't normally would come to the police services for help. In 2014, only 2 cases were registered (excluding those who were not pursued further by families)* – Police Officers, Gaborone City

These data indicate that, though Child Sexual Abuse statistics remain low, CSA is still a problem.

### 5.3 Teenage pregnancy

Teenage pregnancy could be used as a marker for CSA. The narratives from the Participants reflect teenage pregnancy to be a challenge in Botswana. This teenage pregnancy is emphasized in the responses below;

*Sometimes this year [2014] there were around 250 children in Maun area [part of the north-west of Botswana] who are fourteen years and below who are pregnant, and some have children. The question is; how did those children access Health Services without been attended and the abuse reported?*-OVC Technical Advisor, Civil Society.

*I have seen children as young as twelve years pregnant. Those defilement cases, where are they? The system is failing these children. We need to harmonize the laws but also be guided by what is practically happening. The sexual debut is eighteen, but students are falling pregnant at primary school. Our teenage pregnancies have doubled in the last two years. That tells us that there is something we need to do* – Gender Officer, Civil Society.

*It is a problem because our children finish standard seven [final year of primary school] and go to high school and drop out of school in the first few months due to pregnancy. Which shows that this [Child Sexual Abuse] has been going on while still in primary school and only surfaced when in high school. There are different perpetrators such as children abused by other children, adults and so forth* – Primary School Guidance and Counselling Teacher, Letlhakeng Village.

**Figure 4:**
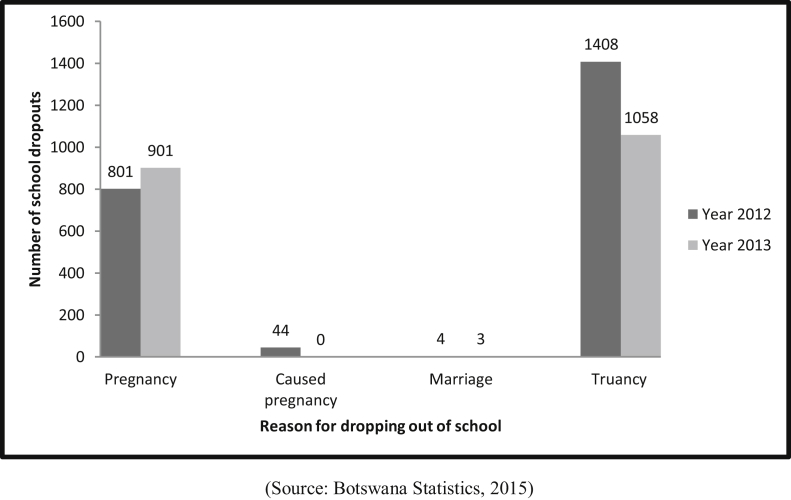
2012–2013 number of secondary school dropouts (N = 175 509).

Figure 4 depicts teenage pregnancy and marriage cases in 2012–2013 among secondary school children. The figures include the boy children who are expelled from school for causing the pregnancy. These pregnancy data are nationwide figures obtained from Education Sector.

(Source: Statistics Botswana, 2012a,b, 2015)

Figure 4 above shows high pregnancy figures that should be considered alongside the low extent of CSA findings presented earlier. For instance, in the year 2013, defilement cases were reported to be 97 in total. Whereas, in the same year, teenage pregnancy rates were more than 8 times (801/97) the defilement case. Additionally, there were marriage cases reported (4 case in 2013 and 3 in 2013) which is also a marker of CSA. Further, truancy figures indicated in the figure above, have been included as some of these children could have dropped out of school due to pregnancy that might not have come to the attention of the authorities. Additionally, children dropping out of school could predispose them to sexual abuse.

**Figure 5:**
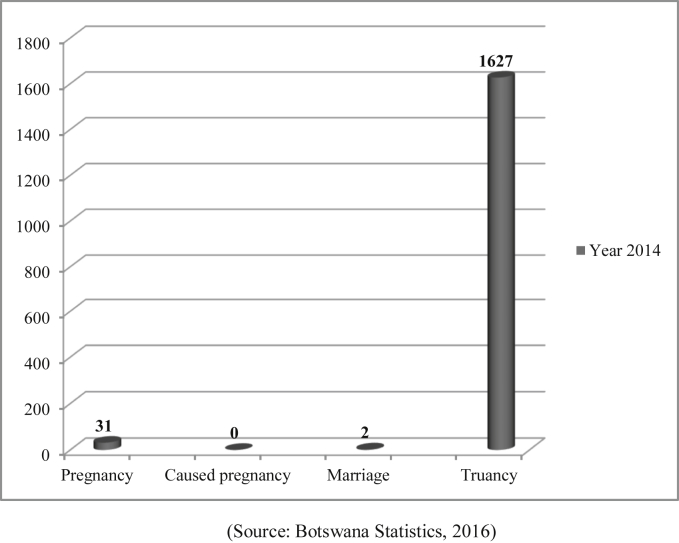
2014 number of primary school dropouts (N = 340 177).

Pregnancy, marriage and trauncy discovered among secondary school children, have also been reported to be occurring at the primary school level as shown in Figure 5. Truancy figures shown in Figure 5 of children who drop out of school for unknown reasons are quite troubling. Some of these truancy cases as mentioned before could be due to pregnancies or marriages.

(Source: Statistics Botswana, 2015)

Furthermore, certain cultures are reported to be perpetuating Child Sexual Abuse. As one participant highlighted;

*It is a serious problem. There are some cultures that perpetuate the problem of Child Sexual Abuse. For instance, if you go to North West [Maun region] early child marriages are rampant. If you just stay in the south [urban], you wouldn't think it [child marriage] was there. Then you start to understand that it is a culture for some people. They do not see anything wrong with it. Again Basarwa [Bushmen] culture is a totally different thing. The moment a child [Bushmen child] is born, the child is given a husband, and it is acceptable. Those [Bushmen] are the ones who need serious education. But you will find that even in customary courts it [child marriage] is normal* –OVC Technical Advisor, Civil Society.

Additionally, out of school children were reported to be at risk of abuse and marriage with Child Protection System not able to offer them services. As narratives from Social Workers reflect;

*It [Child Sexual Abuse] is a big problem. I cover Gaborone Block Six area and have a challenge of children who do not attend school due to their parents' beliefs. This is child abuse. I have six families whose children are not going to school. The challenge with Gaborone Block Six is that I cannot get into houses because they are walled. Families think Jesus Christ is coming and do not want their children contaminated. I have only managed to have access to some families. Foreign families, there are hard to deal with, and I have to involve the police* – Social Services, Gaborone City.

*Professionally I have never come across such cases [child marriages]. But I only hear about them. For instance, the Zezuru [from Zimbabwe] tribe practice that [marry children at a young age]. But we have not really intervened in such cases [child marriage] and other tribes in Botswana. But what I have noticed is that the Zezuru children do not attend school. They would normally attend school up to standard four [middle primary level] when they can read and write. But we haven't really taken those cases seriously* – Social Services, Gaborone City.

One could say, findings that child marriage also occurs, further highlights the seriousness of CSA in Botswana. Some of these children who get pregnant have been reported to create a burden for their parents. As one caregiver indicated;

*Some children have babies at age 11–12 years and then dump those babies on their parents only to become pregnant again. This is grandparent abuse* – Caregiver, Gaborone City.

Compounding establishment of the extent of CSA could be some Participant who reported offering family planning services to some children at fourteen years, in accordance with the National Policy. This was reported by the nurse below.

*The age range for children who come for family planning is 15-19 years. I don't ask questions because the intention is to offer friendly services according to our policy. I had a fourteen-year-old who was brought by the mother because she was already engaging in sex and not using protection. I had to assist her and give her FP [family planning]* – Health Services, Gaborone City.

At the time of my fieldwork, Botswana in collaboration with Center for Disease control embarked on a VACS study which focused on the life experiences of the adolescents (13–24 years) in Botswana. The preliminary findings show that (9.3%) and one in eighteen males (5.5%) among the 18–24 year olds in Botswana experienced sexual violence. These findings are higher than the reported defilement cases reported in 2015 (0.2%). However, the challenge with VACS study is that data were collected from adolescents, excluding very young children who are as affected by CSA as older children.

## 6 Discussion

Failure to establish the extent of CSA coheres previous work in Botswana and other settings (Finkelhor, 1994; Lalor, 2004; Mathoma et al., 2006). Against the backdrop of findings of failure to establish the extent of CSA, it is worthwhile to pause and challenge re-categorization of CSA that is contributing to successful suppression of extent of the problem. This paper argues that CSA is visible to everyone and yet professed to be hidden. Thus, CSA is hidden in plain sight primarily under teenage pregnancy banner.

This finding of high teenage pregnancies would seem to support WHO (2014) estimates that teenage pregnancy is high in Africa especially among the rural poor. Similarly, findings on child marriage are in concordance with UNICEF (2013) as well as WHO (2014) which highlight that, 1 in 4 girls in Africa, marry before 18 despite efforts in place to end child marriage. Furthermore, there is a troublingly high number of children (1408 from secondary school and 1627 from primary school) who dropped out of school in 2013 and 2014 respectively. Studies in Botswana have shown a significant number of children dropping out of school due to sexual harassment (www.unesco.org). Therefore, some of these children who drop out of school without determined causes could be due to CSA in the form of sexual harassment, teenage pregnancy, and child marriage. Further, empirical data shows that education is a protective factor for early pregnancy (WHO, 2014). Therefore, with children dropping out of school, this heightens their vulnerability to CSA which could lead to pregnancy.

All these CSA markers which have been overlooked in Botswana, are caused by multiple factors including children who self-care. The finding in this study of children who self-care would seem to support Ruiz-Casares and Heymann (2009) who found out that, from a socio-historical perspective, Botswana children have been known to self-care. Further, though teenage pregnancy could be better understood from a broader societal context, findings from this study imply the focus on pregnancy rather than the cause of pregnancy. This practice is entrenched in some child laws that offer sexual and reproductive health services to all persons from 12 years (Botswana Adolescent and Reproductive health strategy 2012–2016, 2011). This focus on preventing pregnancy could contribute to CSA considered normative behavior.

Furthermore, teenage pregnancy discourse is used to maintain the invisibility of CSA, diffuse perpetrator responsibility as well as impede the possibility of referring children for appropriate services. This finding would seem to support Pitso and Carmichael (2003) that purport teenage pregnancy to be blamed on children. Therefore, such practices are gender-bound and could reinforce male dominance thesis that holds girls accountable for sex offenses perpetrated against them (Phorano et al., 2005; Phaladze and Tlou, 2006; Purvis and Ward, 2006; Ntsayagae et al., 2008).

Another social norm legitimising CSA is girls' dressing style which is blamed for triggering male sex drive. This finding corroborates with Seloilwe and Thupayagale-Tshweneagae's (2009) finding that, society often blame girls for the dressing style as a reason for sex offenses committed against them. This is seen as drawing from the masculine script that, construct men as unable to control themselves (Seloilwe and Thupayagale-Tshweneagae, 2015).

Further, this male sex drive discourse can become a potential source of power for men and heighten children's vulnerability to sexual abuse. Arguing this finding from the perspective of theories of oppression, this paper contends that CSA could be normalised and maintained. This paper advances the discourse that, CSA is laced within these acts to further victimise children. Analysing these acts is seen as deviating from dominant discourse of shifting the attention from the abuse to the results of abuse. Additionally, critiquing these markers of CSA is seen as advancing the politics surrounding the extent of CSA and therefore, has implications for research, policy and practice.

However, in interpreting teenage pregnancy as a marker of CSA, one could acknowledge the difficulty that practitioners are facing, dealing with children accessing adult services. Narratives from health personnel reflect a tension that exists between offering friendly sexual and reproductive health services to all clients and reporting CSA. The findings reflect priority given to offering friendly services over safeguarding children from CSA. This contradiction is seen as drawing on systems theory as it demonstrates several systemic factors that operate to heighten children's vulnerability.

On the contrary, stakeholders in Botswana could be challenged with difficulty in establishing the extent of CSA as global estimates are also confounded by CSA differing definitions, data collection methods and ages of studied individuals (Finkelhor, 1994; Lalor, 2004).

## 7 Study limitations

Use of secondary data has its own inherent limitations. Since this was a self-funded doctoral research, the use of secondary data was seen an affordable method of data collection. Given another opportunity, I would collect CSA data using a survey. Other than under-reporting which keeps CSA statistics admittedly low, children who reported rape were combined with adults under gender-based violence statistics. This lumping of CSA with adult rape cases compounded adequate use of children's sexual abuse existing data. Furthermore, the sensitivity of Child Sexual Abuse and the taboo surrounding discourse about sexuality in Botswana communities (Makwinja-Morara, 2009; Sabone, 2009), are inherent limitations in this study.

The other limitation was the consenting process in the village which involved the Community Leader (Ramabu, 2019). The involvement of the Community Leader could serve to coerce the villagers to participate in the study.

Lastly, Child Sexual Abuse is still largely hidden from public view and has a tendency to appear normal and this confounds the effort to determine the true picture of CSA (Finkelhor, 1994; Mathoma et al., 2006). However, these limitations do not only serve to highlight the limitations of the study but could be utilized as opportunities for future research.

## 8 Conclusion

This paper has demonstrated that though it was not possible to establish the extent of CSA due to inherent biases in the use of existing data, child sexual abuse in Botswana exists but hidden in plain sight under teenage pregnancy, child marriage, and truancy statistics. This paper is advancing politics surrounding the extent of CSA by challenging obvious markers of CSA and, this could give visibility to CSA that has been successfully suppressed to the detriment of children. However, further research is needed to characterize the scale of teenage pregnancy, child marriage, and reasons for truancy problems that could enable appropriate referral and handling of CSA in Botswana.

## Declarations

### Author contribution statement

N. M. Ramabu: Conceived and designed the experiments; Performed the experiments; Analyzed and interpreted the data; Contributed reagents, materials, analysis tools or data; Wrote the paper.

### Funding statement

This research did not receive any specific grant from funding agencies in the public, commercial, or not-for-profit sectors.

### Competing interest statement

The authors declare no conflict of interest.

### Additional information

No additional information is available for this paper.
